# Supplementary material for: Comparative single-cell transcriptomic analysis of primate brains highlights human-specific regulatory evolution
Source: Nat Ecol Evol. 2023 Sep 4;7(11):1930–43. doi: 10.1038/s41559-023-02186-7 (PMC10627823; doi:10.1038/s41559-023-02186-7)
Supplement: Supplementary file 1 — Supplementary results, references, Figs. 1 to 7 and legends for Tables 1 to 6. [file 41559_2023_2186_MOESM1_ESM.pdf]

nature ecology & evolution

Article

https://doi.org/10.1038/s41559-023-02186-7

Comparative single-cell transcriptomic analysis of primate brains highlights human-specific regulatory evolution

In the format provided by the authors and unedited

Supplementary Results

Evidence for human-specific regulatory rewiring in the ciliopathy gene BBS5

Another gene with potential differential expression regulation specific to the human lineage is *BBS5* (Bardet-Biedl Syndrome 5). Bardet-Biedl Syndrome is a ciliopathic disorder with heterogeneous phenotypes across the human population, including retinal dystrophy, polydactyly, mental retardation, hypogonadism, obesity and kidney dysfunction, and *BBS5* is one of a family of 21 *BBS* genes linked to this disorder (*1*). *BBS5* is expressed in ciliated cells and secretes a protein which is a part of the octameric protein complex (BBSome) required for ciliary membrane biogenesis (*2*), and defects in cilium assembly or function form the basis for human ciliopathies.

Single nucleus profiling reveals significant differences in *BBS5* expression profiles between human and non-human primates, but highly similar profiles across non-human primates. We observe human-specific expression differences not just across 57 homologous cell types (heatmap in **Fig. S4A**), but also within excitatory neurons and non-neuronal cell types (boxplots in **Fig. S4A**).

Given that BBS5 protein is highly conserved across primates (protein sequence similarity of 99.7% in the two great apes; data from Ensembl v107), we hypothesized that expression changes in specific cell types might be driving human-specific divergence. Indeed, we observe human-specific up-regulation of *BBS5* specifically in one layer 5 excitatory neuron cell type and in microglia (**Fig. S4B**). We also observed differential coexpression conservation between humans and other animals in our bulk networks (**Fig. S4C**). Since other *BBS* genes also exhibited distinct expression profiles in humans (see **Fig. S5** for expression divergence in human *BBS1* and *BBS10*), we tested whether *BBS* genes in general showed human-specific regulatory rewiring. Genes related to BBSome formation have been reported to be present across metazoa (*3*). Consistently, we also predict that genes involved in the formation or maintenance of the BBSome complex have conserved function across primates and rodents (**Fig. S4D**), with the exception of *BBS5*.

Cilia have ancient, evolutionarily conserved roles in embryonic development and limb patterning, and are noted as one of the cellular innovations resulting in the emergence of multicellular organisms, but are also known for their structural diversity across species (*4*). Does

the structural diversity translate to network connectivity changes across species? We selected a list of 836 genes involved in cilia morphogenesis and assembly *(*genes from GO terms *GO:0005929, GO:0044782, GO:0060271, GO:0036064)*, and compared their coexpression conservation across five species networks (human, chimp, crab-eating macaque, and two popular disease models - rhesus macaque and mouse). Cilia genes were broadly conserved across species, as expected by their evolutionarily preserved function, but human *BBS5* deviated from this trend (**Fig. S4E**), which suggests both species- and gene-specific regulatory changes. Mouse and zebrafish *BBS* mutant models are known to capture only some but not all phenotypes observed in the human disorder (*5*, *6*). While Bardet-Biedl Syndrome is characterized as a genetic disorder, our results suggest that significantly low coexpression conservation of *BBS5* between humans and other animals could explain the lack of full phenotypic translation between humans and model organisms like mouse and zebrafish. As macaque models become more common to study this ciliopathy, human-specific regulatory divergence could also be related to adverse phenotypes across different tissues in humans and macaques. Further experiments are required to assess the phenotypic impact of this differential coexpression, which could in turn predict the likelihood of translational success for this gene.

Finally, we investigated the role of *cis*-regulatory elements in driving expression variability across human cortical cell types. Candidate *cis*-regulatory elements of *BBS5* (cCREs from ENCODE project) show significant sequence divergence across vertebrates, but are conserved across primates suggesting their primate-specific evolution (**Fig. S6**). Therefore, we switched our focus to search for epigenetic variability across cell types that could be associated with expression variability. Single nucleus ATAC-seq and ChIP-seq profiling of broad cortical cell types in the adult human brain ((*7*), **Fig. S4F**) show both microglia- and oligodendrocyte-specific enhancer activity in BBS5. Single cell ATAC-seq profiling of the developing human brain ((*8*), **Fig. S4G**) only shows microglia-specific enhancer activity in *BBS5*, strongly suggesting a potential mechanism for expression up-regulation in human microglia. Since new phenotypic effects of mutations in *BBS* genes continue to be reported in the literature, we suggest that microglia-specific transcriptional and epigenetic patterns could be implicated in a novel neurodegenerative disease phenotype.

Supplementary References

1. E. Forsythe, J. Kenny, C. Bacchelli, P. L. Beales, Managing Bardet–Biedl Syndrome—Now and in the Future. Front. Pediatr. 6 (2018) (available at https://www.frontiersin.org/article/10.3389/fped.2018.00023).
2. J. B. Li, J. M. Gerdes, C. J. Haycraft, Y. Fan, T. M. Teslovich, H. May-Simera, H. Li, O. E. Blacque, L. Li, C. C. Leitch, R. A. Lewis, J. S. Green, P. S. Parfrey, M. R. Leroux, W. S. Davidson, P. L. Beales, L. M. Guay-Woodford, B. K. Yoder, G. D. Stormo, N. Katsanis, S. K. Dutcher, Comparative Genomics Identifies a Flagellar and Basal Body Proteome that Includes the BBS5 Human Disease Gene. Cell. 117, 541–552 (2004).
3. Y. Nevers, M. K. Prasad, L. Poidevin, K. Chennen, A. Allot, A. Kress, R. Ripp, J. D. Thompson, H. Dollfus, O. Poch, O. Lecompte, Insights into Ciliary Genes and Evolution from Multi-Level Phylogenetic Profiling. Mol. Biol. Evol. 34, 2016–2034 (2017).
4. Z. Carvalho-Santos, J. Azimzadeh, José. B. Pereira-Leal, M. Bettencourt-Dias, Tracing the origins of centrioles, cilia, and flagella. J. Cell Biol. 194, 165–175 (2011).
5. M. R. Bentley-Ford, S. E. Engle, K. R. Clearman, C. J. Haycraft, R. S. Andersen, M. J. Croyle, A. B. Rains, N. F. Berbari, B. K. Yoder, A mouse model of BBS identifies developmental and homeostatic effects of BBS5 mutation and identifies novel pituitary abnormalities. Hum. Mol. Genet. 30, 234–246 (2021).
6. S. Castro-Sánchez, P. Suarez-Bregua, R. Novas, M. Álvarez-Satta, J. L. Badano, J. Rotllant, D. Valverde, Functional analysis of new human Bardet-Biedl syndrome loci specific variants in the zebrafish model. Sci. Rep. 9, 12936 (2019).
7. A. Nott, I. R. Holtman, N. G. Coufal, J. C. M. Schlachetzki, M. Yu, R. Hu, C. Z. Han, M. Pena, J. Xiao, Y. Wu, Z. Keulen, M. P. Pasillas, C. O'Connor, C. K. Nickl, S. T. Schafer, Z. Shen, R. A. Rissman, J. B. Brewer, D. Gosselin, D. D. Gonda, M. L. Levy, M. G. Rosenfeld, G. McVicker, F. H. Gage, B. Ren, C. K. Glass, Brain cell type-specific enhancer-promoter interactome maps and disease risk association. Science. 366, 1134 (2019).
8. R. S. Ziffra, C. N. Kim, J. M. Ross, A. Wilfert, T. N. Turner, M. Haeussler, A. M. Casella, P. F. Przytycki, K. C. Keough, D. Shin, D. Bogdanoff, A. Kreimer, K. S. Pollard, S. A. Ament, E. E. Eichler, N. Ahituv, T. J. Nowakowski, Single-cell epigenomics reveals mechanisms of human cortical development. Nature. 598, 205–213 (2021).
9. R. D. Hodge, T. E. Bakken, J. A. Miller, K. A. Smith, E. R. Barkan, L. T. Graybuck, J. L. Close, B. Long, N. Johansen, O. Penn, Z. Yao, J. Eggermont, T. Höllt, B. P. Levi, S. I. Shehata, B. Aevermann, A. Beller, D. Bertagnolli, K. Brouner, T. Casper, C. Cobbs, R.

Dalley, N. Dee, S.-L. Ding, R. G. Ellenbogen, O. Fong, E. Garren, J. Goldy, R. P. Gwinn, D. Hirschstein, C. D. Keene, M. Keshk, A. L. Ko, K. Lathia, A. Mahfouz, Z. Maltzer, M. McGraw, T. N. Nguyen, J. Nyhus, J. G. Ojemann, A. Oldre, S. Parry, S. Reynolds, C. Rimorin, N. V. Shapovalova, S. Somasundaram, A. Szafer, E. R. Thomsen, M. Tieu, G. Quon, R. H. Scheuermann, R. Yuste, S. M. Sunkin, B. Lelieveldt, D. Feng, L. Ng, A. Bernard, M. Hawrylycz, J. W. Phillips, B. Tasic, H. Zeng, A. R. Jones, C. Koch, E. S. Lein, Conserved cell types with divergent features in human versus mouse cortex. Nature. 573, 61–68 (2019).

10. N. L. Jorstad, J. H. T. Song, D. Exposito-Alonso, H. Suresh, N. Castro, F. M. Krienen, A. M. Yanny, J. Close, E. Gelfand, K. J. Travaglini, S. Basu, M. Beaudin, D. Bertagnolli, M. Crow, S.-L. Ding, J. Eggermont, A. Glandon, J. Goldy, T. Kroes, B. Long, D. McMillen, T. Pham, C. Rimorin, K. Siletti, S. Somasundaram, M. Tieu, A. Torkelson, K. Ward, G. Feng, W. D. Hopkins, T. Höllt, C. D. Keene, S. Linnarsson, S. A. McCarroll, B. P. Lelieveldt, C. C. Sherwood, K. Smith, C. A. Walsh, A. Dobin, J. Gillis, E. S. Lein, R. D. Hodge, T. E. Bakken, Comparative transcriptomics reveals human-specific cortical features (2022), p. 2022.09.19.508480, , doi:10.1101/2022.09.19.508480.
11. Lee, B. T., Barber, G. P., Benet-Pagès, A., Casper, J., Clawson, H., Diekhans, M., Fischer, C., Gonzalez, J. N., Hinrichs, A. S., Lee, C. M., *et al.* (2022) The UCSC Genome Browser database: 2022 update. *Nucleic Acids Res.*, **50**, D1115–D1122.

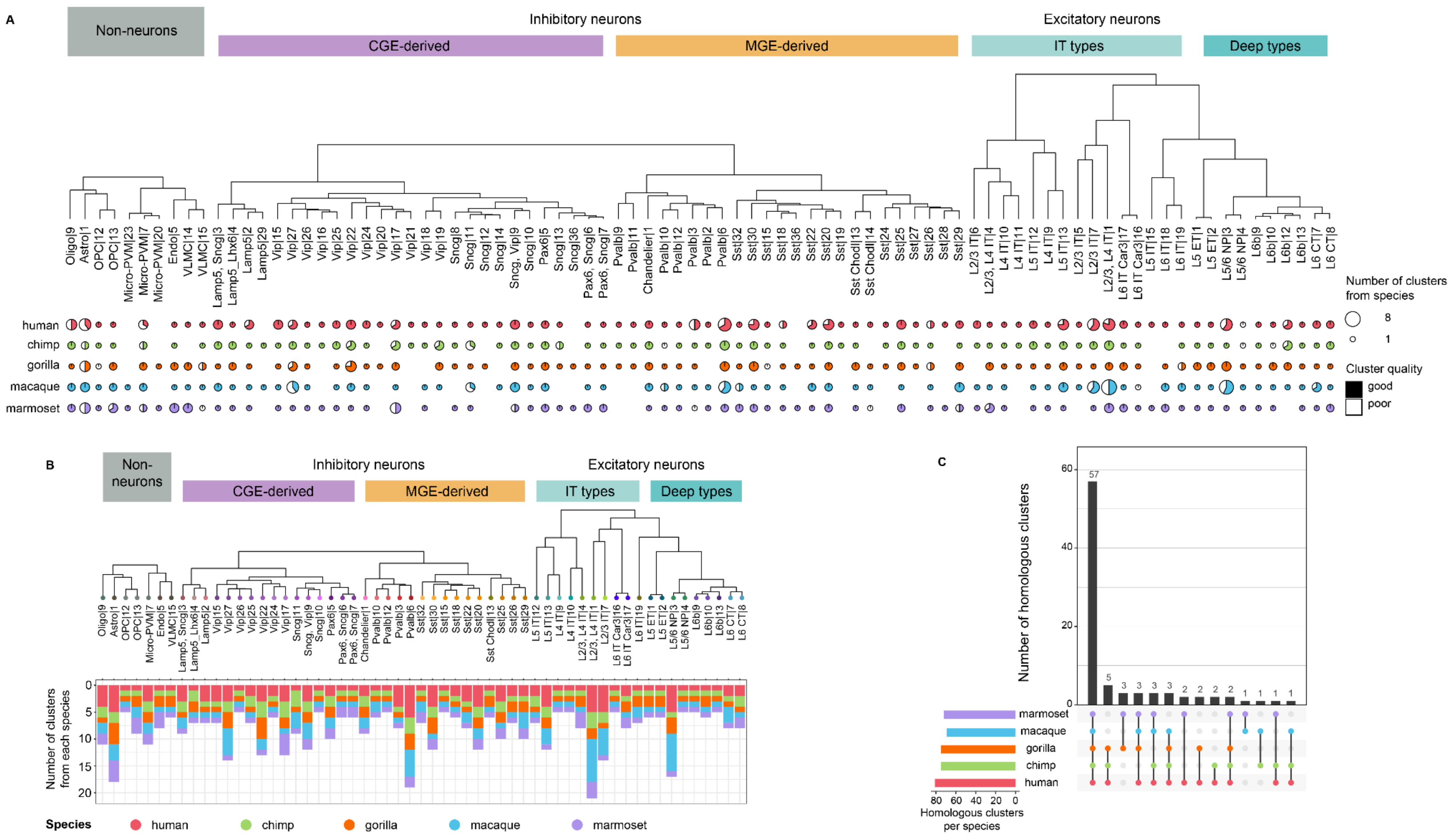

Fig. S1. Constructing consensus taxonomy of homologous cell types. (**A**) Complete cross-species cluster dendrogram generated using average-linkage hierarchical clustering with (1 - average MetaNeighborUS one_vs_all cluster replicability scores) for each pair of 86 cross-species clusters as a measure of distance between cell types. (**B**) Consensus cross-species taxonomy is visualized by pruning the dendrogram (shown in (**A**)) to retain only the 57 homologous cell types shared by all five species. (**C**) Upset plot showing the distribution of 86 homologous cell types across species.

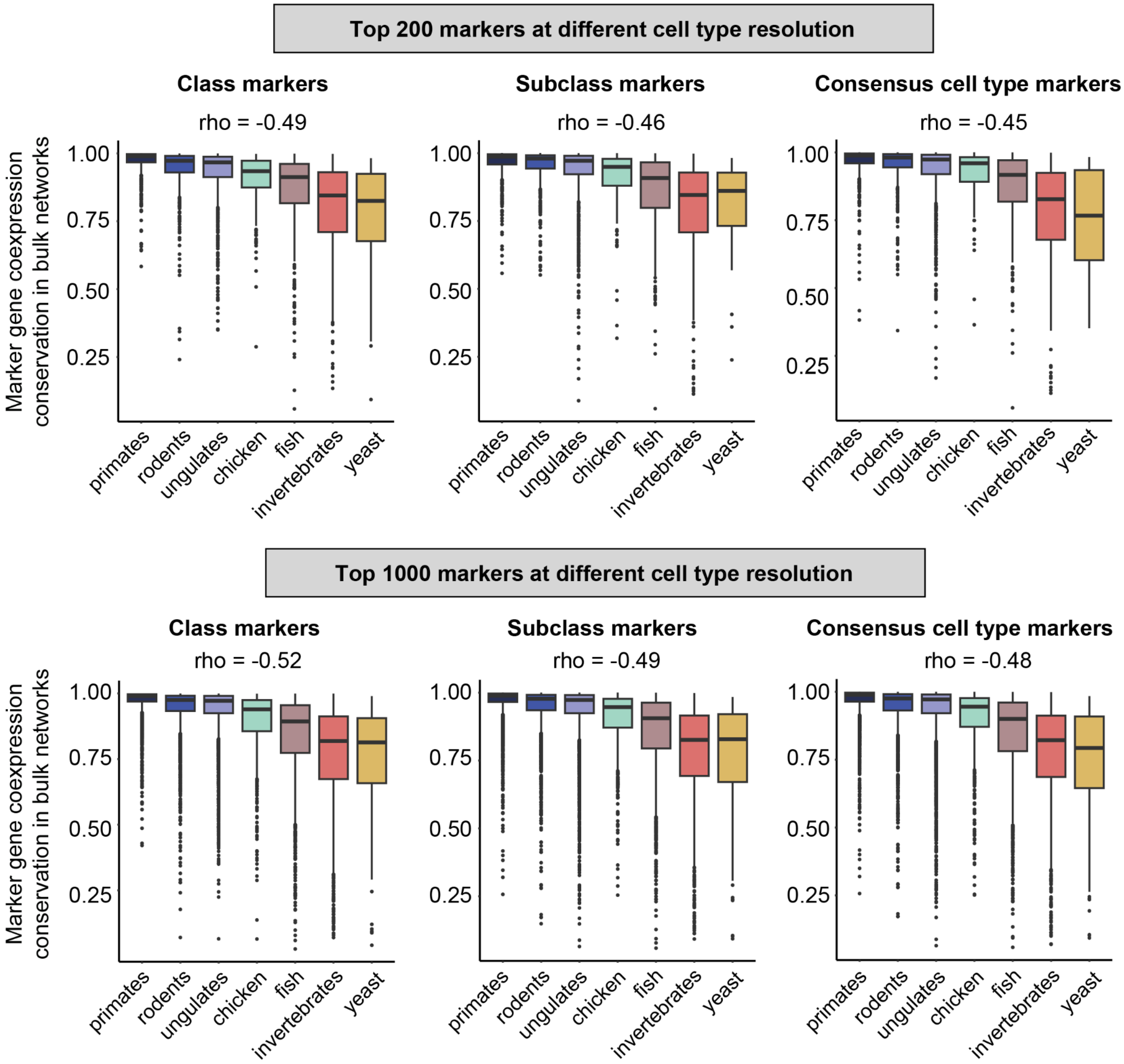

Fig. S2. Gene coexpression conservation across metazoa is robust to marker gene selection criteria. A set of (top) 200 and (bottom) 1000 markers identified from the human MTG atlas at different levels of cell type hierarchy (class, subclass, and consensus cell type) exhibit strong coexpression conservation across 18 other animals. Increasing the size of the marker gene set, and selecting markers at coarse cell type resolution results in a slight decrease in the correlation of marker gene coexpression conservation with phylogenetic distance (indicated by Spearman correlation coefficient in each panel; $P < 2.2e-16$ in all cases). For all boxplots, bounds of the box represent the first and third quartiles, thick line represents the median and whiskers represent 1.5x the interquartile range.

Coexpression conservation across human and mouse aggregated tissue-specific datasets from GEMMA

A

Number of datasets aggregated for each tissue and species

	**human**	**mouse**
**brain**	26	153
**blood**	24	22
**others**	137	235
**all**	187	410

B

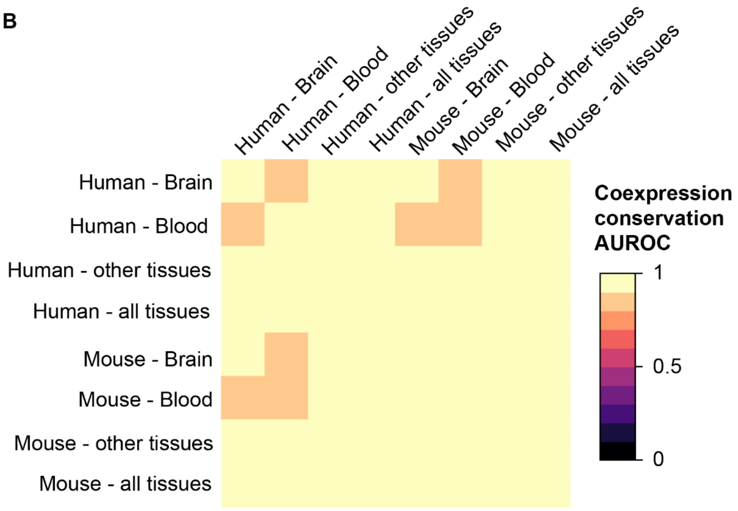

Fig. S3. Tissue heterogeneity in coexpression networks. (**A**) Table lists the number of individual experiments from GEMMA database aggregated to generate brain-specific, blood-specific and other tissue-specific coexpression networks for human and mouse. We also construct a tissue-agnostic network (by aggregating all tissue-specific datasets) to represent the bulk coexpression networks used in **Fig. 4D**. (**B**) Heatmap shows the mean coexpression conservation across tissues and species. Genes have conserved coexpression neighborhoods across different tissues within a species (mean AUROC of 0.98), and across species (mean AUROC of 0.93 between human and mouse datasets), suggesting that our meta-analytic bulk networks capture gene coexpression relationships that are highly replicable across tissues and species.

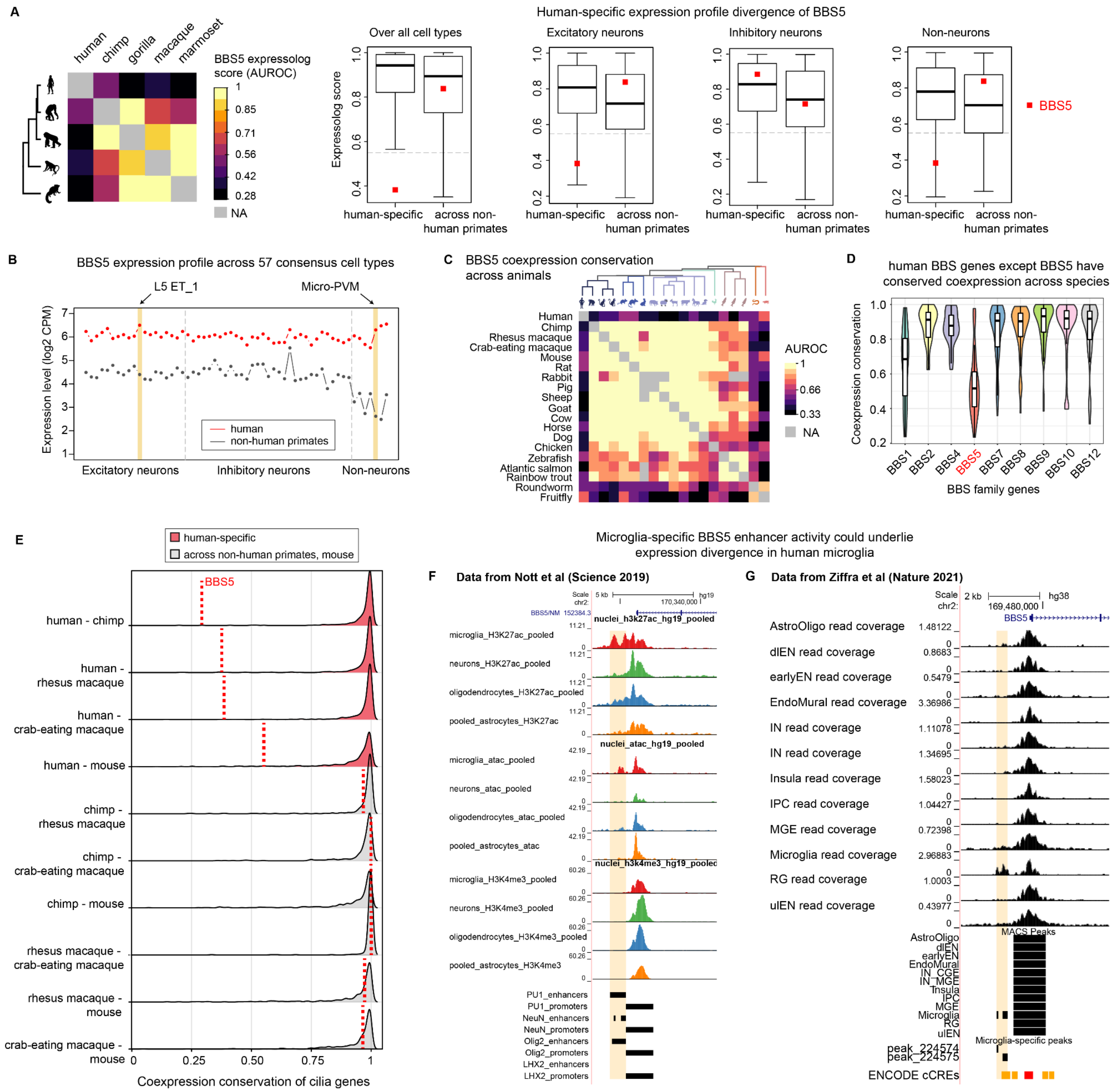

Fig. S4. Regulatory rewiring is associated with human-specific expression profile divergence of *BBS5*. (**A**) (left) Heatmap shows *BBS5* expression profile similarity for each primate pair, and (right) Boxplots show the expression profile similarity distribution for 14,131 orthologs both across and within cell classes, with *BBS5* marked in red. Together, the plots show that *BBS5* has high expression profile similarity between non-human primates, but low similarity between humans and non-human primates (n = 5 species). (**B**) Plot shows the expression profile of *BBS5* in humans with the average expression profile of its ortholog in non-human primates. *BBS5* shows human-specific expression gain in neuronal (layer 5 ET neurons) and non-neuronal (microglia) cell types. (**C**) Heatmap shows high coexpression

conservation specificity of *BBS5* orthologs in all species pairs, excluding humans. Note that the lower scores seen for fish and invertebrates with non-human mammals is consistent with their large evolutionary distances. (**D**) Boxplot shows the distribution of coexpression conservation between human and four closely-related species (chimp, rhesus macaque, crab-eating macaque and mouse) for 9 genes in the *BBS* family, with *BBS5* having the least average score (n = 5 species). (**E**) Distributions of coexpression conservation of genes involved in cilium organization and assembly across primates and mouse indicate their highly conserved function across species, with the exception of *BBS5*, which is conserved across non-human primates and mouse, but diverged only in humans. Single cell epigenomic profiling of broad cortical cell types in the (**F**) adult (*7*), and (**G**) developing human brain (*8*) suggests microglia-specific activity of putative *BBS5* enhancer. While (**F**) also shows oligodendrocyte-specific accessibility for the enhancer, this is not replicated in (**G**). For all boxplots, bounds of the box represent the first and third quartiles, thick line represents the median and whiskers represent 1.5x the interquartile range.

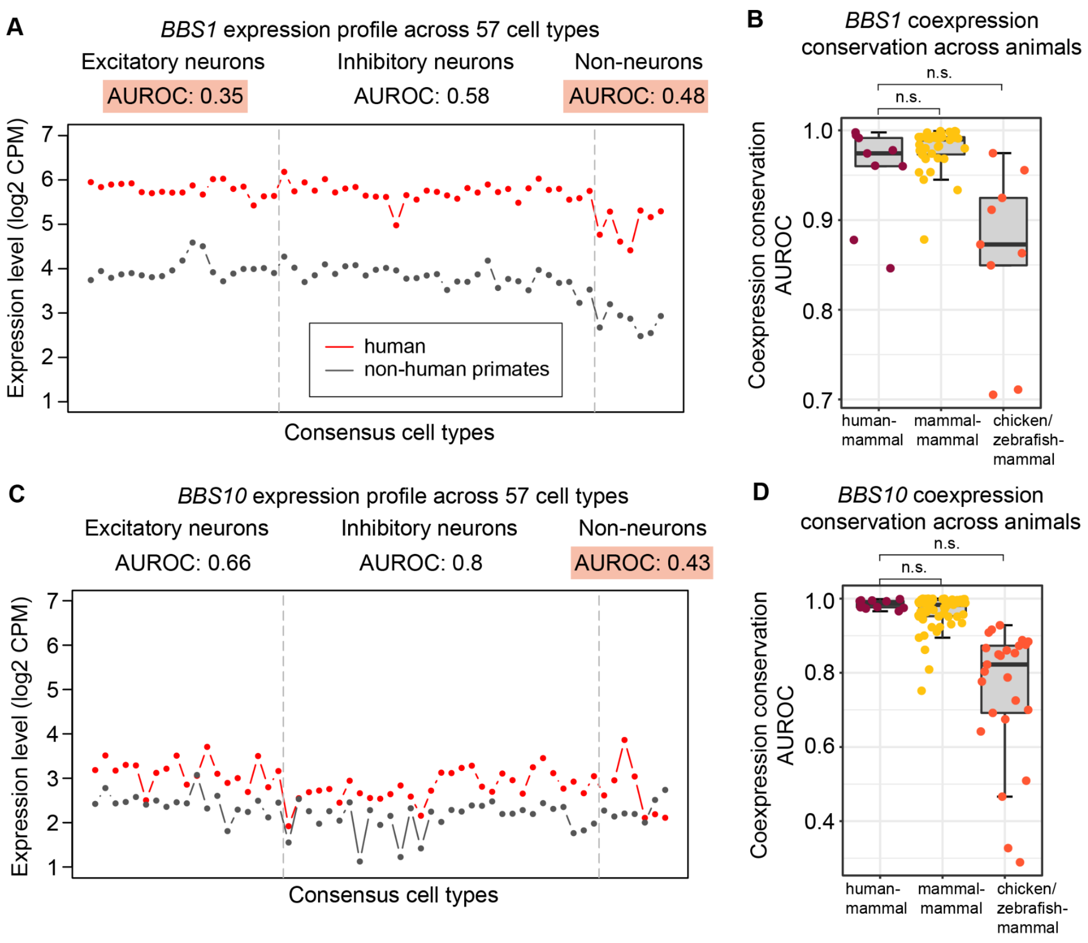

Fig. S5. BBS1 and BBS10 show diverged expression in one or more classes in humans, but have conserved coexpression neighborhoods across metazoa. (**A, C**) Plots compare the expression profile of each gene of interest in humans with the average expression profile of the ortholog in non-human primates. Expressolog scores within each cell class are listed above the plot, and scores < 0.55 are highlighted in orange. (**B, D**) Boxplots show coexpression conservation for orthologs between human and non-human mammals (points colored in maroon, n = 13 species), between pairs of non-human mammals (yellow, n = 12 species), and between non-human mammals and other vertebrates (chicken and zebrafish; orange, n = 14 species). For all boxplots, bounds of the box represent the first and third quartiles, thick line represents the median and whiskers represent 1.5x the interquartile range. For both genes, one-sided Wilcoxon test was performed to evaluate if the coexpression conservation between human and non-human mammals was lower than that between pairs of non-human mammals, and between non-human mammals and other vertebrates. The resulting p-values underwent FDR correction with the Benjamini-Hochberg procedure.

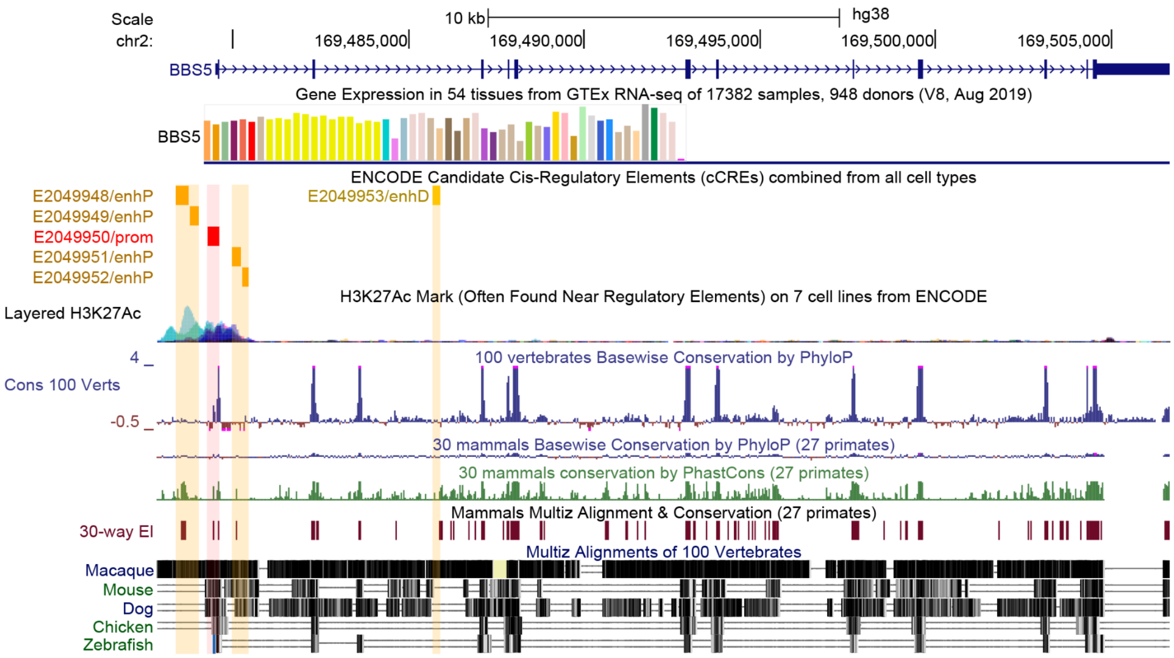

Fig. S6. BBS5 candidate CREs from ENCODE. Genome Browser tracks (*11*) depict high MULTIZ alignment score with macaque and low vertebrate PhyloP scores indicate that putative enhancers regulating human *BBS5* expression are conserved within primates, but diverged across mammals and other vertebrates.

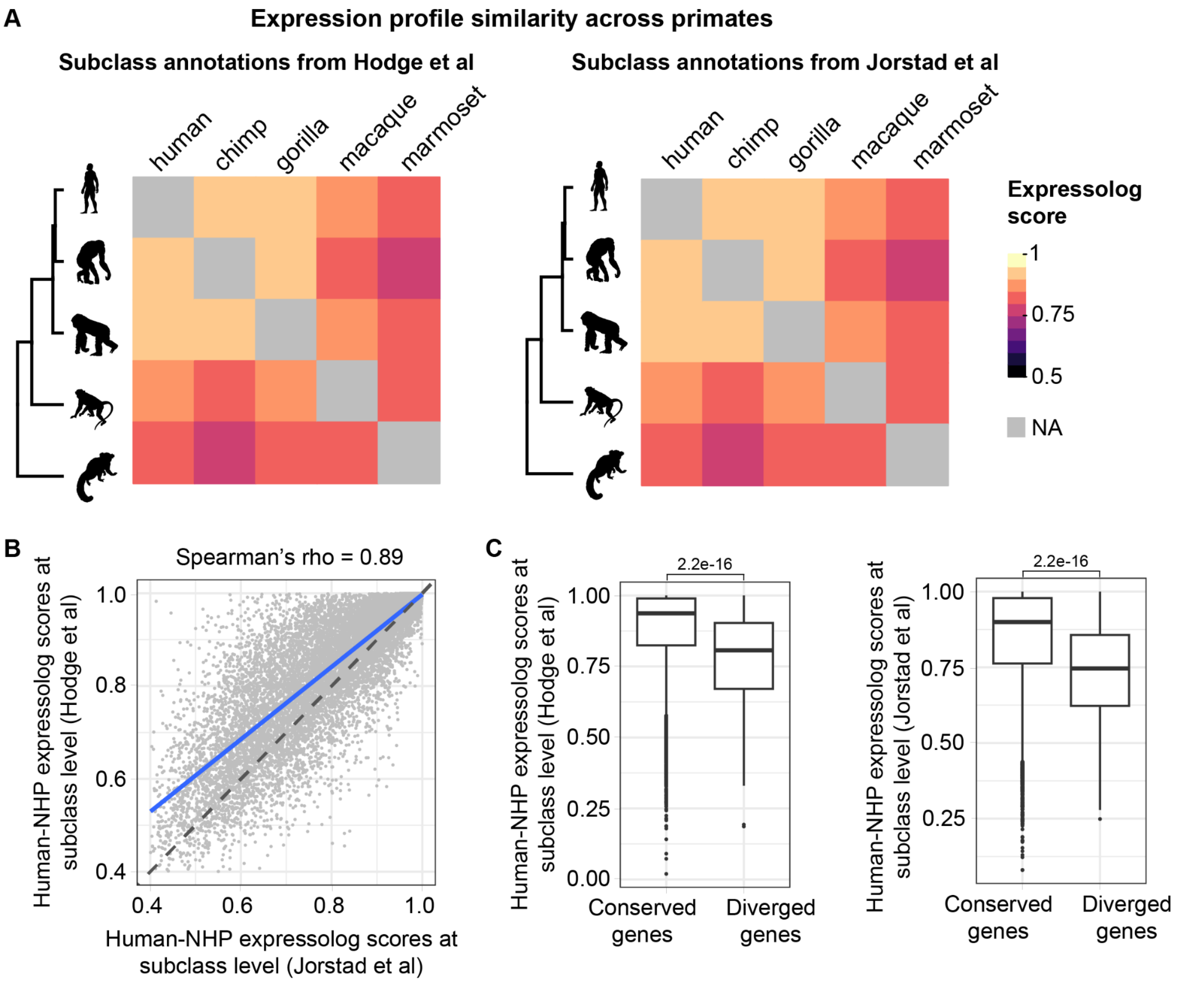

Fig. S7. Expression profile similarity across cell types at the subclass level is robust to different annotation protocols. The primate MTG datasets were re-annotated at the subclass level using the human MTG taxonomy published by Hodge et al (*9*) with an automated annotation method (MetaMarkers). (**A**) Expressolog scores for 14,131 genes were calculated at the subclass level with (left) the subclass labels from Hodge et al, and (right) the subclass labels used in this study (generated by Jorstad et al (*10*)). (**B**) Scatter plot shows the subclass-level expressolog scores between human and non-human primates calculated with labels from Hodge et al and Jorstad et al. Expressolog scores are concordant between both annotation strategies, indicating that the expression profile similarity across cell subclasses is robust to different annotation protocols. (**C**) Boxplots show the distribution of expressolog scores between human and non-human primates for the 139 human-specific "Diverged" genes (reported in **Fig. 5A**), and the remaining "Conserved" genes (two-sided Wilcoxon test P < 2.2e-16 in both cases). Genes diverged in humans had significantly lower expression profile similarity with their non-human primate orthologs irrespective of the subclass labeling method. For all boxplots,

bounds of the box represent the first and third quartiles, thick line represents the median and whiskers represent 1.5x the interquartile range.

Supplementary Tables

Table S1: Summary of single-nucleus transcriptomic datasets from the middle temporal gyrus (MTG) of five primates

Summary of metadata (i.e. number of nuclei, number of donors, sequencing technology and number of within-species clusters) associated with the single-nucleus transcriptomic datasets generated from the middle temporal gyrus from five primates.

Table S2: Performance of 920 HGNC and SynGO gene sets in classifying consensus cell types within and across primates

Classification scores are calculated for gene sets containing 5 or more genes, in each cell type within and across primates. The scores are averaged over species and cell types are reported as 'within_species' and 'cross_species' mean AUROC.

Table S3: List of expressolog score calculated across cell types at different levels of cell hierarchy

Average expressolog scores calculated between human and non-human primates across cell types within each class, subtype and meta-cluster.

Table S4: List of marker genes for cell classes, subclasses and cell types in the human MTG transcriptomic data

Marker genes for 3 cell classes, 24 subclasses and 57 cell types were selected using the MetaMarkers package. We identified 200, 100, 50 genes for each cell class, subclass and cell type respectively, indicated by '1' in the table.

Table S5: Metadata related to cross-species coexpression conservation calculation

List of species and metadata related to their aggregate coexpression networks used to calculate coexpression conservation of 14,131 genes across animal kingdom.

Table S6: 14,131 genes classified into different categories based on their potential for human-specific functional divergence identified using single-cell and bulk transcriptomic data

Genes are classified as:

I. 'Diverged_in_single-cell' if their average expressolog score between human and non-human primates drops below 0.55 in one or more classes

II. 'Diverged_in_bulk' if the distribution of gene coexpression conservation between human and non-human mammals is significantly lower than that within mammals, and between vertebrates (chicken and zebrafish) and non-human mammals

III. 'Diverged_in_both' for 139 genes with concordant divergence in single-cell and bulk data (i.e. I and II are TRUE)

IV. NA, otherwise
