# Supplementary material for: Comparative single-cell transcriptomic analysis of primate brains highlights human-specific regulatory evolution
Source: Nat Ecol Evol. 2023 Sep 4;7(11):1930–43. doi: 10.1038/s41559-023-02186-7 (PMC10627823; doi:10.1038/s41559-023-02186-7)
Supplement: Supplementary file 2 — Reporting Summary [file 41559_2023_2186_MOESM2_ESM.pdf]

nature portfolio

Corresponding author(s): Jesse Gillis

Last updated by author(s): Jul 20, 2023

Reporting Summary

Nature Portfolio wishes to improve the reproducibility of the work that we publish. This form provides structure for consistency and transparency in reporting. For further information on Nature Portfolio policies, see our Editorial Policies and the Editorial Policy Checklist.

Statistics

For all statistical analyses, confirm that the following items are present in the figure legend, table legend, main text, or Methods section.

n/a	Confirmed	
☐	☒	The exact sample size (*n*) for each experimental group/condition, given as a discrete number and unit of measurement
☐	☒	A statement on whether measurements were taken from distinct samples or whether the same sample was measured repeatedly
☐	☒	The statistical test(s) used AND whether they are one- or two-sided<br>*Only common tests should be described solely by name; describe more complex techniques in the Methods section.*
☒	☐	A description of all covariates tested
☐	☒	A description of any assumptions or corrections, such as tests of normality and adjustment for multiple comparisons
☐	☒	A full description of the statistical parameters including central tendency (e.g. means) or other basic estimates (e.g. regression coefficient) AND variation (e.g. standard deviation) or associated estimates of uncertainty (e.g. confidence intervals)
☐	☒	For null hypothesis testing, the test statistic (e.g. *F*, *t*, *r*) with confidence intervals, effect sizes, degrees of freedom and *P* value noted<br>*Give P values as exact values whenever suitable.*
☒	☐	For Bayesian analysis, information on the choice of priors and Markov chain Monte Carlo settings
☒	☐	For hierarchical and complex designs, identification of the appropriate level for tests and full reporting of outcomes
☐	☒	Estimates of effect sizes (e.g. Cohen's *d*, Pearson's *r*), indicating how they were calculated

Our web collection on statistics for biologists contains articles on many of the points above.

Software and code

Policy information about availability of computer code

Data collection	No software was used to collect the data in this study
Data analysis	R version 4, MetaNeighbor version 1.12, MetaMarkers version 0.0.1, STAR version 2.7.7a, RSEM version 1.3.3

For manuscripts utilizing custom algorithms or software that are central to the research but not yet described in published literature, software must be made available to editors and reviewers. We strongly encourage code deposition in a community repository (e.g. GitHub). See the Nature Portfolio guidelines for submitting code & software for further information.

Data

Policy information about availability of data

All manuscripts must include a data availability statement. This statement should provide the following information, where applicable:

- Accession codes, unique identifiers, or web links for publicly available datasets
- A description of any restrictions on data availability
- For clinical datasets or third party data, please ensure that the statement adheres to our policy

Raw sequence data were produced as part of the BRAIN Initiative Cell Census Network and are available for download from the Neuroscience Multi-omics Archive (https://assets.nemoarchive.org/dat-net1412), and the relevant file locations are listed in https://github.com/hamsinisuresh/Primate-MTG-coexpression. Integrated snRNA-seq gene expression dataset and associated metadata for each primate species are available on the BICCN Human/NHP website. Data used in the analyses reported in this study can be accessed from https://labshare.cshl.edu/shares/gillislab/resource/Primate_MTG_coexp/, and from Figshare (DOI: https://

doi.org/10.6084/m9.figshare.22032104). Details on the processing and clustering of primate MTG snRNA-seq datasets are available at: https://github.com/AllenInstitute/Great_Ape_MTG. Code to reproduce the cross-species expression profile similarity and coexpression conservation analysis can be accessed from https://github.com/hamsinisuresh/Primate-MTG-coexpression.

Human research participants

Policy information about studies involving human research participants and Sex and Gender in Research.

Reporting on sex and gender	N/A
Population characteristics	N/A
Recruitment	N/A
Ethics oversight	N/A

Note that full information on the approval of the study protocol must also be provided in the manuscript.

Field-specific reporting

Please select the one below that is the best fit for your research. If you are not sure, read the appropriate sections before making your selection.

[x] Life sciences [ ] Behavioural & social sciences [ ] Ecological, evolutionary & environmental sciences

For a reference copy of the document with all sections, see nature.com/documents/nr-reporting-summary-flat.pdf

Life sciences study design

All studies must disclose on these points even when the disclosure is negative.

Sample size	Sample size was not predetermined. To generate the primate MTG atlas, single nuclei were isolated from postmortem brains of human (n = 7), chimpanzee (n = 7), gorilla (n = 4), macaque (n = 3), and marmoset (n = 3). This allowed us to collect over 570,000 nuclei from high quality specimens across five primates that passed stringent quality control, and generated consistent transcriptomic clusters across donors.
Data exclusions	No data were excluded from the analysis
Replication	Cluster replicability within and across species was assessed using MetaNeighbor. Cell type clusters within each species were highly reproducible across donors and sequencing technologies. Cell types defined at the class, subclass, and consensus cluster levels were highly replicable across primates.
Randomization	Randomization is not possible since the categorical variable of interest is "species"
Blinding	Blinding of samples is not possible since species-specific properties need to be accounted for in alignment and comparative transcriptomic analysis

Reporting for specific materials, systems and methods

We require information from authors about some types of materials, experimental systems and methods used in many studies. Here, indicate whether each material, system or method listed is relevant to your study. If you are not sure if a list item applies to your research, read the appropriate section before selecting a response.

Materials & experimental systems

n/a	Involved in the study	
[x]	[ ]	Antibodies
[x]	[ ]	Eukaryotic cell lines
[x]	[ ]	Palaeontology and archaeology
[x]	[ ]	Animals and other organisms
[x]	[ ]	Clinical data
[x]	[ ]	Dual use research of concern

Methods

n/a	Involved in the study	
[x]	[ ]	ChIP-seq
[x]	[ ]	Flow cytometry
[x]	[ ]	MRI-based neuroimaging
